# FKBP51 and FKBP52 regulate androgen receptor dimerization and proliferation in prostate cancer cells

**DOI:** 10.1002/1878-0261.13030

**Published:** 2021-06-19

**Authors:** Keisuke Maeda, Makoto Habara, Mitsuyasu Kawaguchi, Hiroaki Matsumoto, Shunsuke Hanaki, Takahiro Masaki, Yuki Sato, Hideyasu Matsuyama, Kazuki Kunieda, Hidehiko Nakagawa, Midori Shimada

**Affiliations:** ^1^ Department of Biochemistry Joint Faculty of Veterinary Science Yamaguchi University Japan; ^2^ Graduate School of Pharmaceutical Sciences Nagoya City University Japan; ^3^ Department of Urology Graduate School of Medicine Yamaguchi University Ube Japan

**Keywords:** immunophilin, peptidyl‐prolyl cis/trans isomerase, prostate cancer, steroid receptor

## Abstract

The growth of prostate cancer is dependent on the androgen receptor (AR), which serves as a ligand‐specific transcription factor. Although two immunophilins, FKBP51 and FKBP52, are known to regulate AR activity, the precise mechanism remains unclear. We found that depletion of either FKBP51 or FKBP52 reduced AR dimer formation, chromatin binding, and phosphorylation, suggesting defective AR signaling. Furthermore, the peptidyl‐prolyl cis/trans isomerase activity of FKBP51 was found to be required for AR dimer formation and cancer cell growth. Treatment of prostate cancer cells with FK506, which binds to the FK1 domain of FKBPs, or with MJC13, an inhibitor of FKBP52–AR signaling, also inhibited AR dimer formation. Finally, elevated expression of FKBP52 was associated with a higher rate of prostate‐specific antigen recurrence in patients with prostate cancer. Collectively, these results suggest that FKBP51 and FKBP52 might be promising targets for prostate cancer treatment through the inhibition of AR dimer formation.

## Graphical abstract

We demonstrated that the two immunophilins, FKBP51 and FKBP52, are required for dimer formation and chromatin binding of AR. The PPIase activity of FKBP51 is essential for cancer cell proliferation and AR dimer formation. We conclude that both FKBPs promote dimer formation in AR and activate AR‐dependent transcription, which is associated with the etiology of prostate cancer.

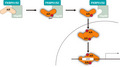

**Abbreviations**
AR: androgen receptor
Co‐IP: co‐immunoprecipitation
FKBP51: FK506‐binding protein 5
FKBP52: FK506‐binding protein 4
Hsp90: heat shock protein 90
PPIase: peptidyl‐prolyl cis/trans isomerase
PSA: prostate‐specific antigen
TCGA: The Cancer Genome Atlas

## 1 Introduction

The androgen receptor (AR) is a ligand‐dependent nuclear receptor that is required for the expression of androgen‐regulated genes. AR is key to normal prostate development as well as oncogenesis of prostate cancer, especially the development of castration‐resistant prostate cancer [1]. Androgen deprivation therapy suppresses the progression of hormone‐sensitive prostate cancer through the inhibition of AR signaling, although prostate cancer often acquires resistance to androgen deprivation therapy [2].

Androgen receptor activity is regulated in a highly coordinated dynamic manner at multiple levels such as expression, androgen binding, nuclear translocation, homodimer formation, and DNA binding of AR. Androgen binding induces conformational changes, including intramolecular interactions in the AR, which facilitates its translocation to the nucleus [3]. In the nucleus, an AR dimer binds to androgen‐responsive elements in the regulatory regions of its target genes [4].

Androgen receptor is localized to the cytosol in the absence of androgen, as a complex with the molecular chaperone heat shock protein 90 (Hsp90), and various other cochaperones, including the small acidic protein p23, FKBP (FK506‐binding protein) 51, and FKBP52 [5, 6, 7]. FKBPs are a large family of proteins that possess peptidyl‐prolyl cis/trans isomerase (PPIase) activity. The immunosuppressants FK506 and rapamycin bind to FKBP, resulting in the inhibition of its PPIase activity [8, 9].

FKBP51 and FKBP52 are homologous proteins with 54.7% identity and 88.1% similarity in their amino acid sequences, in addition to a similar structural organization. These proteins contain an NH_2_‐terminal FK1 domain that is responsible for its PPIase activity and an FK1‐like FK2 domain that lacks PPIase activity. FK506 binds to the FK1 domain and inhibits its enzymatic activity, but does not bind to the FK2 domain. The COOH‐terminal region contains three tandem repeats known as the tetratricopeptide repeat (TPR) domain, which mediates interactions with other proteins, including the COOH terminus of Hsp90 [10].

Despite the sequence and structural similarity between the two FKBPs, these have been shown to exert distinct and diverse effects on various steroid receptors. FKBP51 inhibits the activity of glucocorticoid receptor (GR) and progesterone receptor (PR) by reducing their ligand binding activity [6, 11]. However, the effect of FKBP51 on AR differs from that on GR and PR: FKBP51 promotes binding of androgen to AR, thereby increasing its transcriptional activity [12].

Instead, FKBP52 has been shown to contribute to the regulation of steroid receptors by enhancing hormone binding, stabilizing the steroid receptors, and promoting their translocation into the nucleus from the cytoplasm [5, 6, 13, 14, 15], although a study with FKBP52‐deficient mouse embryonic fibroblasts has shown that FKBP52 is dispensable for the binding of androgen to AR or for its nuclear translocation [16].

The PPIase domain, but not its activity, is responsible for interaction with steroid receptors and is essential for the receptor activity [6, 17]. Several previous studies have demonstrated the role of FKBP51 and FKBP52 in AR signaling; yet, little is known about the precise mechanism by which FKBP51 and FKBP52 control AR activity.

In the present study, we deciphered how FKBP51 and FKBP52 regulate AR activity. In contrast to the regulation of GR and mineralocorticoid receptor (MR), we found that both FKBPs were not required for AR translocation to the nucleus, but required for dimer formation and chromatin binding of AR. In addition, the PPIase activity of FKBP51 was essential for cancer cell proliferation as well as AR dimer formation. We conclude that FKBP51 and FKBP52 promote dimer formation of AR and activate AR‐dependent transcription, which is associated with the etiology of prostate cancer.

## 2 Methods

### 2.1 Cell culture and reagents

22Rv1 (CRL‐2502; ATCC, Manassas, VA, USA), LNCaP (CRL‐1740; ATCC), LNCaP AI (CRL‐3314; ATCC), PC3 (CRL‐1435; ATCC), and 5637 (HTB‐9; ATCC) cells were cultured in RPMI 1640 (187‐02705; Wako, Osaka, Japan). DU145 (HTB‐81; ATCC), TCCSUP (HTB‐5; ATCC), UMUC3 (CRL‐1749; ATCC), and J82 (HTB‐1; ATCC) cells were cultured in EMEM (055‐08975; Wako). T24 (HTB‐4; ATCC) and RT4 (HTB‐2; ATCC) cells were cultured in McCoy's 5A medium (16600‐082; Gibco, Dublin, Ireland). SW 780 (CRL‐2169; ATCC) cells were cultured in Leibovitz's L‐15 medium (128‐06075; Wako). RPE1 cells (gift from M. Nakanishi, Tokyo University) were cultured in DMEM/F‐12 (11330‐032; Gibco). MJ‐90 (gift from M. Nakanishi) and HEK293T (632180; Takara, Kusatsu, Shiga, Japan) cells were cultured in DMEM (044‐29765; Wako). All cells were cultured in medium supplemented with 10% FBS (173012; SIGMA, Saint Louis, MO, USA) and antibiotics (15240062; Thermo Fisher Scientific, Waltham, MA, USA). All cells were cultured at 37 °C under 5% CO_2_.

Cells were treated with DHT (A8380‐1G; Sigma‐Aldrich, Saint Louis, MO, USA), FK506 (069–06191; Wako), SAFit1 (HY‐102079; MedChemExpress, Monmouth Junction, NJ, USA), SAFit2 (HY‐102080; MedChemExpress), and MJC13 (SML0347; Sigma‐Aldrich). DHT was dissolved in methanol (MeOH), while FK506 and MJC13 were dissolved in DMSO. DHT was used at a concentration of 10 nm. FK506, SAFit1, SAFit2, and FKBP51 PPIase inhibitors were used at the indicated concentrations. SAFit2 was used at 10 μm for 24 h in the NanoBiT assay.

### 2.2 Construction of short hairpin RNA

To generate lentivirus‐based shRNA constructs, a 21‐base shRNA‐coding fragment with an ACGTGTGCTGTCCGT loop was introduced into pENTR4‐H1 digested with BglII. The pENTR4‐H1‐shRNA vectors were incubated with CS‐RfA‐ETBsd or CS‐RfA‐ETHyg vectors and Gateway™ LR™ Clonase Enzyme Mix (Invitrogen, Dublin, Ireland) for 2 h at 25 °C, which produced the CS‐RfA‐ETBsd‐shRNA vector. The target sequences for the lentivirus‐based shRNA were FKBP51‐1: GGAAGATAGTGTCCTGGTTAG, FKBP51‐2: GGAACAGACAGTCAAGCAATG, FKBP52‐1: GCGGAATCATTCGCAGAATAC, and FKBP52‐2: GCAAGGACAAATTCTCCTTTG. If not stated otherwise, shFKBP51‐1 or shFKBP52‐1 was used for the experiments.

### 2.3 Transfection

Lentivirus generation and infection were performed as described previously [18]. Lentiviruses expressing the respective genes were generated by cotransfection of 293T cells with lentiviral‐packaging vectors (1.54 μg of Pax2 and 0.86 μg of pMD2) and 2.0 μg of the respective CS‐RfA‐shRNA or FKBP, using 6.6 μg of PEI MAX**^®^** (24765–1; Polysciences Inc., pH 7.0, Warrington, PA, USA). Two days after transfection, the virus‐containing supernatant was collected and filtered. Cells were then transduced with each lentivirus in the presence of polybrene (1 : 1000) in standard culture media for 24 h. Cells infected with viruses were treated with 1 μg·mL^−1^ puromycin (P7255; Sigma‐Aldrich), 10 μg·mL^−1^ blasticidin (022–18713; Wako), or 200 μg·mL^−1^ hygromycin (H3274; Sigma‐Aldrich) for 2 days. To express the inducible gene, Dox (D9891; Sigma‐Aldrich) was added to the medium at a concentration of 1 μg·mL^−1^. For transient overexpression of AR‐HA and V5‐AR plasmids, cells were transfected with the corresponding plasmid using polyethylenimine (PEI; Polysciences Inc., pH 7.0). Briefly, cells at 50–70% confluence were incubated for 24 h with 6 μL of PEI and 3 μg of DNA. DNA–lipid complexes were diluted in Opti‐Minimal Essential Medium (Opti‐MEM; Gibco) and incubated for 30 min, before being added to the cells. After transfection, the cell medium was changed to a serum medium. Cells were analyzed 48 h after transfection.

### 2.4 Cell growth

To determine cell growth, 5 × 10^4^ LNCaP cells and 4 × 10^4^ 22RV1 cells were plated in a 3.5 cm culture dish. The day after seeding was considered as day 0, on which the medium was replaced with serum‐free medium containing Dox. The number of cells that attached to the dish was counted after trypsin treatment.

### 2.5 Synthesis of FKBP51 PPIase inhibitor

The FKBP51 PPIase inhibitor was synthesized in accordance with a previous report [19], with slight modifications (Figs S1 and S2). ^1^H‐ and ^13^C‐NMR spectra of the final product (the FKBP51 PPIase inhibitor) completely matched the report. Detailed procedures and spectral data are shown in Figs S1 and S2.

### 2.6 Colony formation assay

A colony formation assay was carried out to evaluate the effect of the FKBP51 PPIase inhibitor on the proliferation of the cancer cells. Cells (1 × 10^3^) were seeded into a 6 cm dish. After adhesion, the cells were treated with different concentrations of the FKBP51 PPIase inhibitor. The clones developed were fixed with formaldehyde and stained using 0.4% Trypan blue (207‐03252; Wako).

### 2.7 Immunoblotting

Collected cells were washed with ice‐cold PBS, suspended in sample buffer (2% SDS, 10% glycerol, 100 μm DTT, 0.01% BPB, and 50 mm Tris/HCl at pH 6.8), and boiled for 5 min. The total cell lysates obtained were subjected to subcellular fractionation using the Subcellular Protein Fractionation Kit for Cultured Cells (78840; Thermo Scientific, Waltham, MA, USA). Raw digital images were captured using a ChemiDoc Imaging System (Bio‐Rad, Hercules, CA, USA). The bands of the target protein were quantified using imagej (Rockville, MD, USA) and normalized with that of β‐Actin, unless otherwise indicated. The following antibodies were used in this study: AR (cs5153; Cell Signaling Technology, Danvers, MA, USA), AR‐pS81 (07‐1375; Millipore, Burlington, MA, USA), β‐Actin (ab6276; Abcam, Cambridge, UK), FKBP51 (ab126715; Abcam), FKBP52 (10655‐1‐AP; Proteintech, Rosemont, IL, USA), FoxA1 (sc101058; Santa Cruz Biotechnology, Dallas, TX, USA), H3 (ab1791; Abcam), HA (PAB10343; Abnova, Taipei City, Taiwan), HSP90 (sc13119; Santa Cruz Biotechnology), α‐Tubulin (T5168; Sigma‐Aldrich), V5 (R960‐25; Thermo Fisher Scientific), anti‐mouse IgG HRP (NA9310; GE Healthcare, Chicago, IL, USA), and anti‐rabbit IgG HRP (NA9340; GE Healthcare).

### 2.8 Mutagenesis

LgBiT‐AR P767A, AR P767A‐SmBiT, and pENTR1A‐FKBP52 F67Y were generated using the PrimeSTAR Mutagenesis Basal Kit (R046A; Takara). The primers used in this study are as follows: AR‐P767A F: CTTCGCCGCTGATCTGGTTTTCAATG, AR‐P767A R: AGATCAGCGGCGAAGTAGAGCATCCT, FKBP52 F67Y F: AAAGTACGACTCCAGTCTGGATCGCAAG, and FKBP52 F67Y R: CTGGAGTCGTACTTTGTGCCATCTAATAG.

### 2.9 Plasmid

Using pcDNA3 as the parental vector, the coding region of the AR gene was fused to ‐HA or V5‐ using the In‐Fusion^®^ HD Cloning Kit (Clontech, Kusatsu, Shiga, Japan). pENTR4‐H1‐FKBP WT or mutant vectors were incubated with CS‐RfA‐ETBsd vectors and Gateway™ LR™ Clonase Enzyme Mix (Invitrogen) for 2 h at 25 °C, which resulted in the CS‐RfA‐ETPuro‐FKBP vector. Fusion protein expression constructs [fusion of AR (or FKBP or HSP90) and LgBiT (or SmBiT)] were prepared by directional cloning the full ORF region of these genes into NanoBiT vectors (pFN33K LgBiT TK‐neo Flexi Vector, pFC34K LgBiT TK‐neo Flexi Vector, pFN35K SmBiT TK‐neo Flexi Vector, and pFC36K SmBiT TK‐neo Flexi Vector; Promega, Madison, WI, USA), according to the manufacturer's instructions.

### 2.10 Immunoprecipitation analysis

Total proteins (500 μg) were precleared with protein‐G Sepharose (17061801; GE Healthcare) for 1 h at 4 °C and immunoprecipitated with 0.02 μg of anti‐FKBP51, anti‐FKBP52, or immunoglobulin G (as a control; 12‐270; Sigma‐Aldrich) overnight at 4 °C. For co‐immunoprecipitation (Co‐IP), 1.1 μg anti‐V5 was used against 300 μg of protein. The immune complexes were recovered with protein‐G Sepharose for 1 h and then washed with IP Kinase buffer (IPK: 50 mm HEPES, 150 mm NaCl, 2.5 mm EGTA, 1 mm EDTA, 1 mm DTT, 0.1% Tween‐20, 10% glycerol, 50 mm NaF, 0.1 mm Na_3_Vo_4_, 15 mm PNPP, 80 mm β‐glycerophosphate) at least three times, centrifuged, and subjected to SDS/PAGE, followed by immunoblotting.

### 2.11 Real‐time PCR

Total RNA extraction was performed as described previously [20]. A total of 50 ng RNA was reverse‐transcribed with random primers using the High‐Capacity cDNA Reverse Transcription Kit (4368814; Applied Biosystems, Waltham, MA, USA). Quantitative real‐time PCR was performed using FastStart™ Universal SYBR**^®^** Green Master (42917900; Roche, Basel, Switzerland) and an StepOnePlus™ real‐time PCR system (Applied Biosystems). Expression levels were normalized to those of β‐Actin. Primers used for the real‐time PCR included AR‐F: AGCAGCAGGGTGAGGATG and AR‐R: GACTGCGGCTGTGAAGGT.

### 2.12 Immunofluorescence

LNCaP cells were grown on coverslips and treated with DHT or MeOH for 20 h. Cells were pretreated with 1 μg·mL^−1^ Dox in a medium containing charcoal‐stripped FBS for 2 days. After treatment, the cells were fixed in ice‐cold MeOH, followed by incubation with 3% acetone for 10 min at −20 °C. Cells were then washed three times with PBS and incubated with 0.5% Triton/PBS for 10 min, followed by washing and blocking in 5% goat serum for 30 min, before incubation with antibodies (at a dilution of 1 : 200) for an hour to detect AR. Antigens were visualized using anti‐rabbit antibodies coupled to FITC (1 : 200; 1 h). Photomicrographs were taken at a magnification of 400× using DeltaVision Elite (GE Healthcare).

### 2.13 NanoBiT assay

One day before transfection, HEK293T cells were seeded at a density of 2 × 10^4^ cells/well in a 96‐well plate containing MEM‐α with charcoal‐stripped FBS. On the following day, expression vectors and PEI were diluted in 16 μL of Opti‐MEM (Gibco). Of note, 6.25 ng·μL^−1^ of LgBiT‐AR and AR‐SmBiT or LgBiT‐AR P767A and AR P767A‐SmBiT expression vectors were used for the AR dimer formation assay. Further, AR‐LgBiT and FKBP‐SmBiT; LgBiT‐HSP90 and SmBiT‐FKBP52; AR‐LgBiT; and LgBiT‐FKBP51, AR‐LgBiT, and SmBiT‐HSP90 expression vectors were used for the protein–protein interaction assays. Diluted vectors and PEI were combined and vortexed and then incubated for 30 min at room temperature. After incubation, the solution mixture was directly added to the cells drop‐wise. Two days after transfection, the luminescence was measured using a Nano‐Glo^®^ Live Cell Assay System (Promega) and Nivo (PerkinElmer, Waltham, MA, USA) or ARVO X4 (PerkinElmer) or GloMax^®^ Navigator (Promega). DHT or MeOH was added 4 h before or 15 min after the addition of the luminescence reagent.

### 2.14 Luciferase reporter assay

The pcDNA3 AR and pCMV Rluc (Renilla luciferase) constructs were provided by M. Okada. 293T cells were cultured at a density of 2 × 10^4^ cells/well in 96‐well plates for 24 h in MEM Alpha (41061‐029; Gibco) containing 5% charcoal‐stripped FBS and 1 μg·mL^−1^ Dox. PC3 cells were cultured at a density of 1 × 10^4^ cells/well in 96‐well plates for 24 h in RPMI Medium 1640 (11835‐030; Gibco) containing 5% charcoal‐stripped FBS and 1 μg·mL^−1^ Dox. The cells were transfected with PSA‐430‐pGL3 [21], pcDNA3 AR, which contained an activated‐AR‐inducible Fluc sequence, and pCMV Rluc using Lipofectamine3000 Reagent (Invitrogen). Twenty‐four hours after transfection, the cells were treated with 10 nm DHT or DMSO for 24 h. The luminescence caused by firefly luciferase and Renilla luciferase was measured using the Dual‐Glo Luciferase Assay System (E2920; Promega) and Nivo (PerkinElmer). Fold induction was calculated as the ratio of the luminescence of firefly luciferase to that of Renilla luciferase.

### 2.15 TCGA dataset analysis

Transcriptome data were obtained from the TCGA Pan‐Cancer dataset using the University of California at Santa Cruz Cancer Genomics Browser (http://xena.ucsc.edu/). We selected only primary tumor samples with expression data (*n* = 9858). Batch effect‐normalized mRNA data were used as the expression value. Box plots were generated using graphpad prism 6 (GraphPad Software, San Diego, CA, USA).

### 2.16 Tissue samples

We performed immunohistochemical (IHC) analysis with antibodies against FKBP52 for specimens obtained from prostate cancer patients as reported previously [22]. A total of 50 newly diagnosed prostate cancer tissues from radical prostatectomies were obtained from the Yamaguchi University Hospital (Ube, Japan) between 2000 and 2016. We used serum PSA levels after radical prostatectomy as a surrogate end‐point, with a level ≥ 0.2 ng·mL^−1^ designated as PSA failure. PSA recurrence‐free survival rate was determined as the percentage of patients without PSA failure. The experiments were undertaken with the understanding and written consent of each subject. The study methodologies conformed to the standards set by the Declaration of Helsinki. The study methodologies were approved by the local ethics committee.

### 2.17 Immunohistochemistry

Formalin‐fixed and paraffin‐embedded tissue specimens were subjected to H&E staining and IHC staining as described in previous study [22]. For each sample, 3 μm thick sections were deparaffinized in xylene, dehydrated in ethanol, and incubated in a 0.3% hydrogen peroxide solution in MeOH for 10 min at room temperature. The sections were then microwaved in a 0.01 m citrate‐buffered solution (pH 6.0) for 15 min and covered in blocking solution (IMMUNO SHOT; Cosmo Bio Co. Ltd., Tokyo, Japan) for 30 min at room temperature. Following that, the sections were incubated overnight at 4 °C with a primary antibody (anti‐FKBP52 antibody, EPR6619, 1 : 200 dilution; Abcam), according to the manufacturer's instructions, followed by incubation with the respective secondary antibody (N‑Histofine Simple Stain™ MAX PO (MULTI); cat. no. 414152F; Nichirei Biosciences Inc., Tokyo, Japan) for 30 min at room temperature. The *H*‑score was used to evaluate IHC staining in the present study. Briefly, > 500 tumor cells were counted in five different fields of vision in each section (×100 magnification) and the *H*‑score was calculated by multiplying the percentage of positive cells by the intensity (strongly stained, 3×; moderately stained, 2×; and weakly stained, 1×), yielding a possible range of 0–300. Two independent examiners (HM and HM) judged the scores, and the mean score obtained was set as the representative score. Cutoff for the *H*‑score was determined based on the receiver operating characteristic curve.

### 2.18 Statistical analysis

Association with PSA failure‐free probability was determined using Kaplan–Meier curves, while a log‐rank test was used to determine the level of significance. The relationship between the two data groups was compared using Tukey's multiple comparison test or Student's *t*‐test with graphpad prism 6 (GraphPad Software). A *P‐*value of < 0.05 was considered statistically significant.

## 3 Results

### 3.1 FKBP51 and FKBP52 contribute to the proliferation of prostate cancer cells

**Fig. 1:**
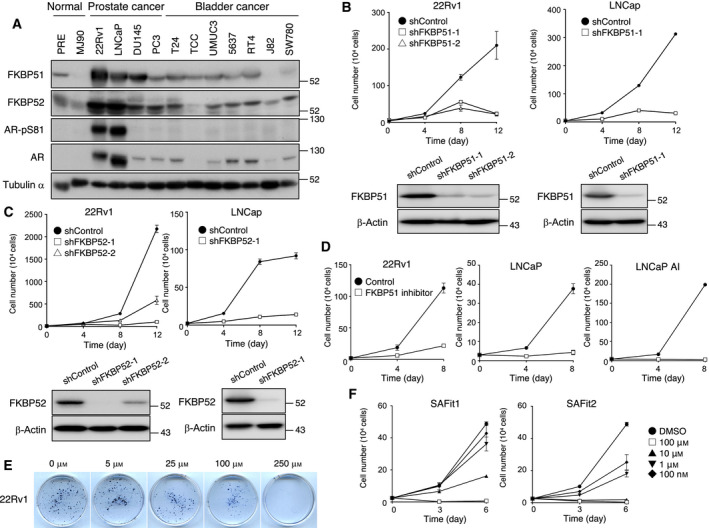
FKBP51 and FKBP52 are involved in prostate cancer cell growth. (A) Immunoblotting of FKBP51 and FKBP52 in normal and cancer cell lines. (B, C) Cells expressing Dox‐inducible shControl (luciferase), shFKBP52 (B), or shFKBP51 (C) were cultured in the presence of Dox for the indicated number of days. Cells were collected, followed by counting of the cell numbers. Each experimental point is the average of the quantitation of four aliquots, expressed as mean ± SEM. Immunoblots of the cells used in the experiment are shown at the bottom. The cells were cultured in the presence of Dox for 4 days. (D) 22Rv1, LNCaP, and LNCaP AI cells were cultured in the presence of FKBP51 PPIase inhibitor or DMSO for the indicated number of days. Cells were collected, followed by counting of the cell numbers. The data have been expressed as mean ± SEM of three independent experiments. (E) 22Rv1 cells were cultured in the presence of different concentrations of FKBP51 PPIase inhibitor. The colonies were visualized using Trypan blue staining. (F) 22Rv1 cells were cultured in the presence of different concentrations of SAFit1 or SAFit2, or DMSO for the indicated number of days. Cells were collected and then counted. The data have been expressed as mean ± SEM of three independent experiments.

Steroid receptors are known to form large oligomeric structures with chaperones and cochaperones, including Hsp90, p23, and TPR‐domain proteins, such as FKBP51 and FKBP52. FKBPs, a large family of immunophilins that are conserved in eukaryotes, mediate diverse cellular functions including protein folding, cellular signaling, and transcription [23]. To elucidate the regulatory mechanism of AR by FKBP51 and FKBP52, we examined the expression of FKBP51 and FKBP52 in normal cells as well as multiple prostate cancer and bladder cancer cell lines (Fig. 1A). We found that the expression of FKBP51 and FKBP52 in AR‐positive prostate cancer cell lines was greater than that in normal cells and bladder cancer cells. Given that FKBP51 has been shown to contribute to the proliferation of prostate cancer cells [12], we examined whether FKBP52 is required for cancer cell growth. For this purpose, we depleted FKBP52 using a lentivirus‐delivered short hairpin RNA (shRNA) that allows conditional knockdown upon addition of doxycycline (Dox). Depletion of FKBP52 substantially attenuated cell proliferation in two independent AR‐positive prostate cancer cells, 22Rv1 and LNCaP (Fig. 1B). We confirmed that FKBP51 was also required for the proliferation of these cells (Fig. 1C), consistent with previous studies [24, 25]. These results suggest that FKBP51 and FKBP52 are essential for the proliferation of these prostate cancer cells.

It has been reported that the PPIase activity of FKBP52 is dispensable for AR activity [15]. We next examined whether the enzymatic activity of FKBP51 is required for the proliferation of 22Rv1 cells. We synthesized a compound that specifically binds to the FK1 domain of FKBP51 and inhibits its PPIase activity [19]. Notably, this FKBP51 PPIase inhibitor attenuated the proliferation and colony formation of 22Rv1 cells (Fig. 1D,E). In addition, we investigated whether this inhibitor has an effect only on cancer cells that grow in an androgen‐dependent manner. We found that the PPIase inhibitor suppressed the proliferation of both androgen‐dependent (LNCaP) and androgen‐insensitive prostate cancer cells (LNCap AI) (Fig. 1D), suggesting that the PPIase activity of FKBP51 is essential for cell proliferation in addition to AR regulation. We confirmed that SAFit1 and SAFit2, commercially available FKBP51 inhibitors, also attenuated the proliferation of 22Rv1 cells (Fig. 1F). These results suggest that the inhibition of PPIase activity of FKBP51 might be a promising approach for prostate cancer treatment.

### 3.2 FKBP51 and FKBP52 do not affect the expression and localization of AR

**Fig. 2:**
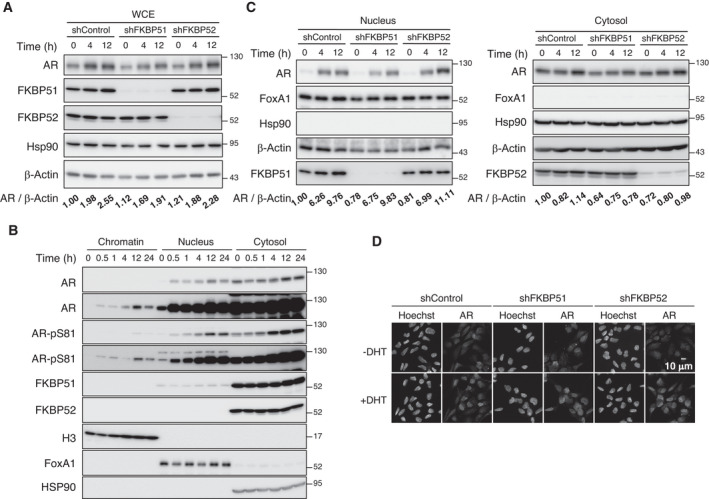
FKBP51 and FKBP52 are not involved in the expression and localization of AR. (A) 22Rv1 cells expressing Dox‐inducible shControl, shFKBP51, or shFKBP52 were cultured in the presence of Dox for 2 days. The cells were collected after treatment with or without 10 nm DHT for 4 or 12 h, following which the total cell lysates were analyzed using immunoblotting with the indicated antibodies. The relative band intensity of AR was normalized to that of β‐Actin. The relative ratios of AR/β‐Actin have been mentioned at the bottom (*n* = 3). (B) 22Rv1 cells were treated with 10 nm DHT for the indicated time periods, followed by subcellular fractionation (*n* = 3). Samples were analyzed using immunoblotting with the indicated antibodies. Histone H3, FOXA1, and Hsp90 were used as the markers for chromatin, nuclear, and cytosol, respectively. (C) 22Rv1 cells expressing Dox‐inducible shControl, shFKBP51, or shFKBP52 were prepared as mentioned in (A) and (B) (*n* = 3). Nuclear (left) and cytosolic (right) fractions were analyzed using immunoblotting with the indicated antibodies. (D) Immunofluorescence of AR in LNCaP cells expressing shRNA. Cells were treated with DHT or MeOH as a control (−DHT) for 20 h (*n* = 3). Hoechst 33342 staining and expression of AR have been shown. Scale bar: 10 μm.

Although FKBP51 and FKBP52 have been shown to contribute to the regulation of AR signaling, the detailed underlying mechanisms still remain unclear. We examined the expression of AR in 22Rv1 cells depleted of FKBP51 or FKBP52 at 0, 4, and 12 h after the onset of dihydrotestosterone (DHT) treatment. While the expression of AR increased upon DHT binding in control cells, consistent with previous studies that have demonstrated that AR protein stability increases upon DHT binding [26, 27], depletion of FKBP51 or FKBP52 had no significant effect on AR expression, suggesting that FKBP51 and FKBP52 do not affect the expression of AR (Fig. 2A).

**Fig. 3:**
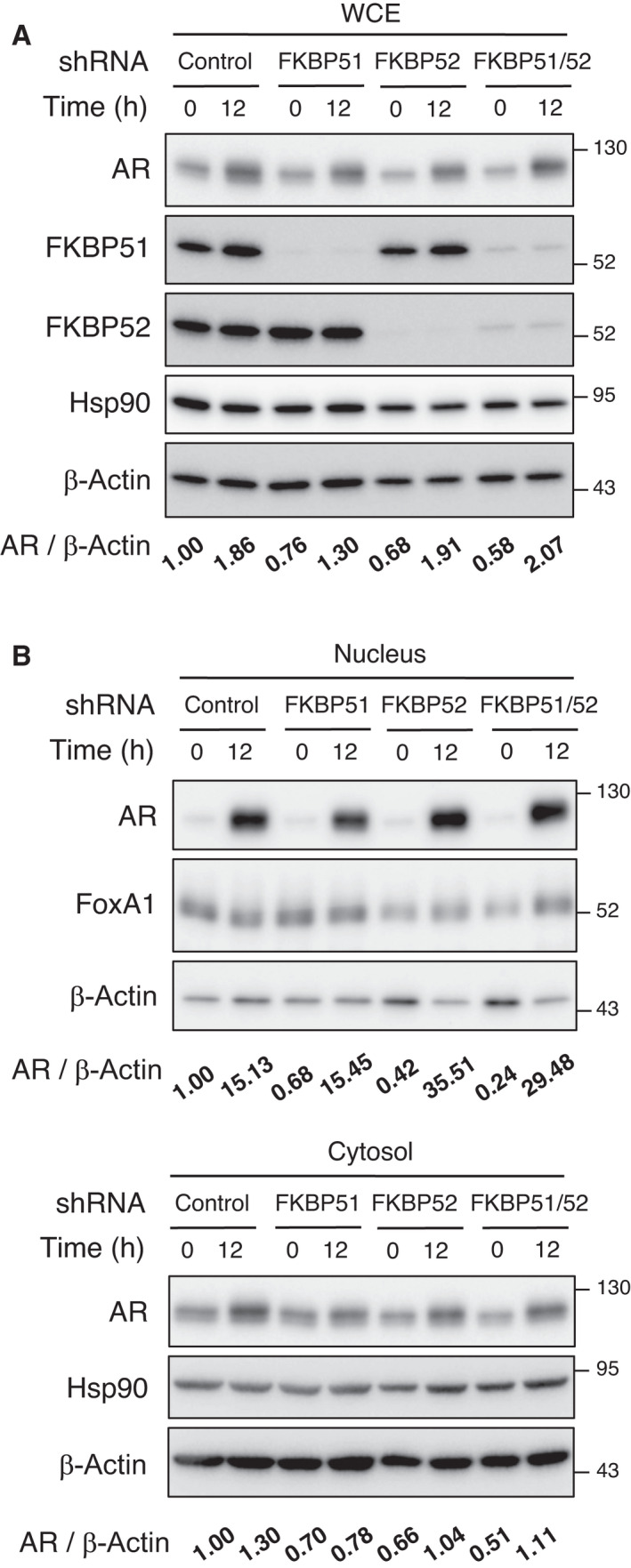
Expression and nuclear localization of AR are not affected by FKBP51 or FKBP52 depletion. (A) 22Rv1 cells expressing Dox‐inducible shControl, shFKBP51, or/and shFKBP52 were cultured in the presence of Dox for 2 days. The cells were collected after treatment with or without 10 nm DHT for 12 h, following which the total cell lysates were analyzed using immunoblotting with the indicated antibodies. The relative band intensity of AR was normalized to that of β‐Actin. The relative ratios of AR/β‐Actin are shown at the bottom (*n* = 3). (B) 22Rv1 cells expressing Dox‐inducible shControl, shFKBP51, or/and shFKBP52 were prepared as mentioned in Fig. 2A,B. Nuclear and cytosolic fractions were analyzed using immunoblotting with the indicated antibodies (*n* = 3).

Androgen receptor is known to translocate from the cytosol to the nucleus in response to ligand binding, and FKBP52 has been shown to contribute to nuclear localization of multiple nuclear receptors [14, 28]. Subcellular fractionation of 22Rv1 cell lysates revealed that AR translocated to the nucleus in response to DHT treatment, with phosphorylation of serine at position 81 of AR (AR‐Ser^81^), which is associated with AR activity (Fig. 2B). Of note, the nuclear translocation of AR in FKBP51‐ or FKBP52‐depleted cells did not differ from that in control cells (Fig. 2C). In addition, immunofluorescence analysis with antibodies against AR confirmed that the DHT treatment‐induced nuclear translocation of AR was not significantly affected upon depletion of FKBP51 or FKBP52 in 22Rv1 cells (Fig. 2D). Furthermore, we found that the expression and nuclear translocation of AR did not change in either single or double knockdown cells (Fig. 3A,B). These results suggest that FKBP51 and FKBP52 do not affect the expression and localization of AR.

### 3.3 Depletion of FKBP51 or FKBP52 attenuates AR dimer formation

**Fig. 4:**
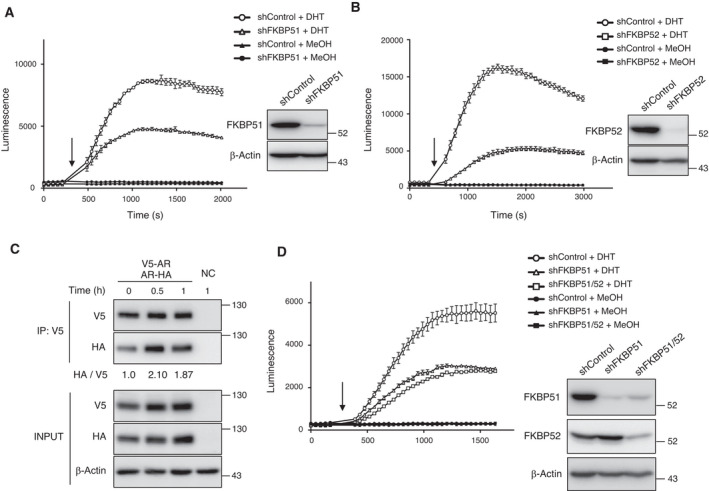
FKBP51 and FKBP52 are essential for AR dimer formation. (A, B) Dimer formation of AR was determined using NanoBiT analysis in HEK293T cells expressing shRNA. LgBiT‐AR and AR‐SmBiT expression vectors were cotransfected into HEK293T cells expressing Dox‐inducible shControl, shFKBP51, or shFKBP52. Cells were cultured in the serum‐starved condition in the presence of Dox for 48 h and treated with 10 nm DHT or MeOH as a control. The arrow indicates the time‐point of addition of DHT or MeOH. The luminescence was monitored by Nivo (PerkinElmer), values for which have been expressed as mean ± SEM of triplicate experiments. Immunoblots of cells subjected to NanoBiT analysis are shown on the right. Cells were cultured in the presence of Dox for 2 days. (C) Co‐IP of AR‐HA and V5‐AR. HEK293T cells transiently expressing AR‐HA and V5‐AR were cultured in the serum‐starved condition in the presence of Dox for 48 h and treated with or without 10 nm DHT. The cells were collected after culturing for the indicated times, following which the cell lysates were incubated with α‐V5 antibodies to precipitate AR. Coprecipitated proteins were analyzed by immunoblotting with α‐HA or α‐V5. The HA relative density toward V5 was measured using image lab Software (Hercules, CA, USA). NC–negative control sample. The relative ratios of HA/V5 have been mentioned at the bottom. (D) Dimer formation of AR was determined using NanoBiT analysis in HEK293T cells expressing shRNA. HEK293T shControl, shFKBP52, or shFKBP51/52 cells were assayed as in A. The arrow indicates the time‐point of addition of DHT or MeOH. The luminescence was monitored by Nivo (PerkinElmer), values for which have been expressed as mean ± SEM of triplicate experiments. Immunoblots of cells subjected to NanoBiT analysis are shown on the right. Cells were cultured in the presence of Dox for 2 days.

Ligand binding promotes homodimer formation of steroid hormone receptors, including AR, and activates transcription of target genes. Therefore, we examined AR dimerization in living 22Rv1 cells using the Nano‐Luc Binary Technology (NanoBiT) system and found that AR forms dimers immediately after the addition of DHT (Fig. 4A,B). To verify ligand‐induced AR dimerization using another independent approach, we transfected HEK293T cells with two expression vectors for AR tagged with V5 or HA. The cell lysates were prepared at 0, 0.5, and 1 h after the onset of DHT treatment. Co‐IP analysis revealed that AR dimerization was promoted upon DHT treatment (Fig. 4C). Of note, the luminescence emission induced by AR dimer formation was reduced 0.54‐fold in FKBP51‐deficient cells and 0.30‐fold in FKBP52‐deficient cells compared to the highest signal value in shControl cells (Fig. 4A,B). We confirmed the deficiency of AR dimerization in FKBP51‐ or FKBP52‐depleted cells using distinct shRNA targets (Fig. S1). These results suggest that both FKBP51 and FKBP52 are essential for ligand‐induced dimerization of AR. In addition, we found that deficiency of AR dimerization in cells depleted of both FKBP51 and FKBP52 was not significantly different from that of FKBP51‐depleted cells (Fig. 4D). These results suggest that both FKBP51 and FKBP52 contribute to AR signaling via the same pathway.

**Fig. 5:**
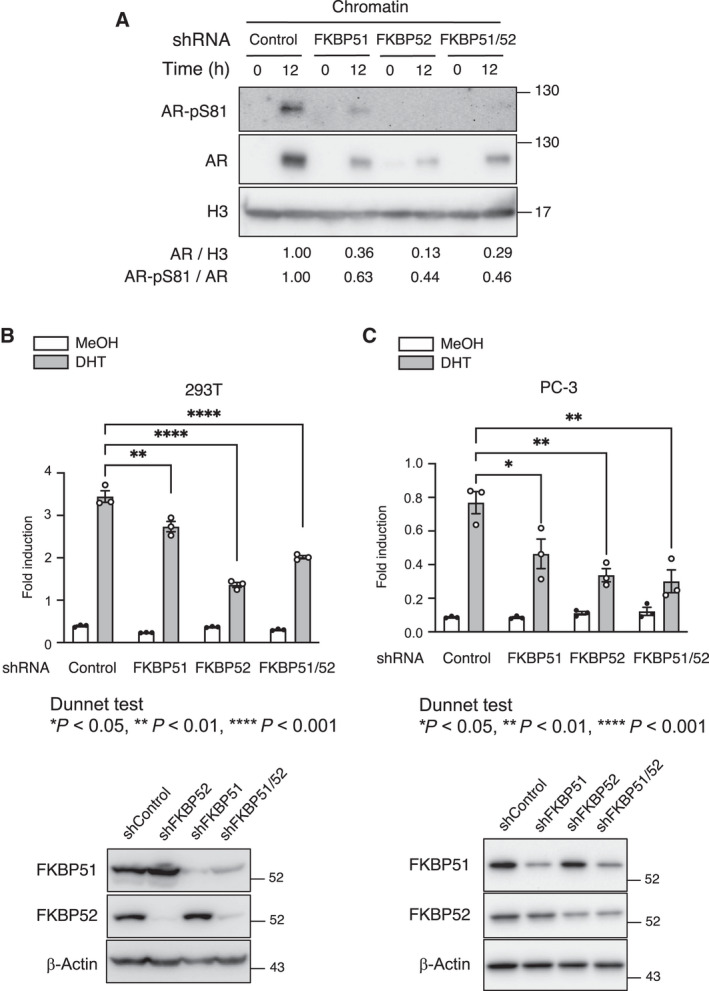
Transcriptional activity of AR is reduced in FKBP51‐ or FKBP52‐depleted cells. (A) 22Rv1 cells expressing Dox‐inducible shControl, shFKBP51, or/and shFKBP52 were cultured in the serum‐starved condition for 48 h and treated with 10 nm DHT for 12 h. Cells were collected for subcellular fractionation, following which the chromatin fractions were subjected to immunoblotting using the indicated antibodies. The relative band intensities of AR and AR‐pS^81^ were normalized to those of H3 and AR, respectively. The relative ratios of AR/β‐Actin or AR‐pS^81^/AR are shown at the bottom (*n* = 3). (B) HEK293T cells transfected with PSA‐430‐pGL3, pCMV Rluc, and expression plasmids for AR were treated with MeOH (0 nm) or DHT (10 nm) for 24 h. Firefly luciferase activities were normalized with Renilla luciferase activities. Values are the mean ± SEM (*n* = 3). **P < 0.01 and ****P < 0.001 (Dunnett's test vs DHT‐treated shControl expressed group). Immunoblots of cells subjected to Luciferase reporter assay are shown on the right. Cells were cultured in the presence of Dox for 2 days. (C) PC‐3 cells transfected with PSA‐430‐pGL3, pCMV Rluc, and expression plasmids for AR were treated with MeOH (0 nm) or DHT (10 nm) for 24 h. Firefly luciferase activities were normalized with Renilla luciferase activities. Values are the mean ± SEM (*n* = 3). *P < 0.05, **P < 0.01 (Dunnett's test vs DHT‐treated shControl expressed group). Immunoblots of cells subjected to Luciferase reporter assay are shown at the bottom. Cells were cultured in the presence of Dox for 2 days.

Furthermore, there was an increase in chromatin‐bound AR and active form of AR (AR‐Ser^81^) upon DHT treatment in shControl 22Rv1 cells, but the extent was significantly decreased in FKBP51‐ or/and FKBP52‐depleted cells (Fig. 5A), indicating insufficient activation of AR in these cells. DHT‐induced AR activity, as measured by reporter gene assays, was significantly reduced in FKBP51‐ or FKBP52‐depleted PC3 and 293T cells (Fig. 5B,C). We further confirmed that double knockdown cells did not enhance the deficiency of AR activity compared to the single knockdown cells. Together, these results suggest that both FKBP51 and FKBP52 are required not only for AR dimerization but also for its transcriptional activity.

### 3.4 The PPIase activity of FKBP51 contributes toward AR dimerization

**Fig. 6:**
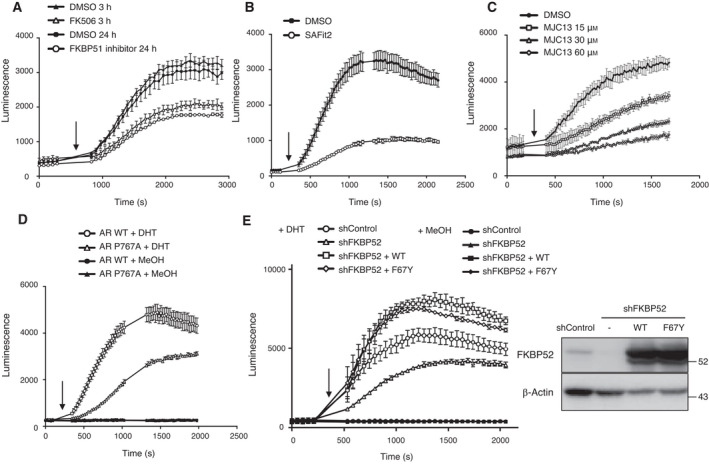
The involvement of PPIase activities in AR dimerization. (A) Dimer formation of AR was determined using NanoBiT analysis in HEK293T cells. LgBiT‐AR and AR‐SmBiT expression vectors were cotransfected into HEK293T cells. The cells were treated with 50 μm FK506 for 3 h or 250 μm FKBP51 PPIase inhibitor for 24 h. The arrow indicates the time‐point of addition of DHT or MeOH. The luminescence was monitored by ARVO X4 (PerkinElmer), values for which have been expressed as mean ± SEM of triplicate experiments. (B) Dimer formation of AR was determined using NanoBiT analysis in HEK293T cells. Cells were treated with the indicated 10 μm SAFit2 for 24 h. The arrow indicates the time‐point of addition of DHT. The luminescence was monitored by Nivo (PerkinElmer), values for which have been expressed as mean ± SEM of triplicate experiments. (C) Dimer formation of AR was determined using NanoBiT analysis in HEK293T cells. LgBiT‐AR and AR‐SmBiT expression vectors were cotransfected into HEK293T cells. Cells were treated with the indicated concentration of MJC13 for 3 h. The arrow indicates the time‐point of addition of DHT or MeOH. The luminescence was monitored by GloMax^®^ Navigator (Promega), values for which have been expressed as mean ± SEM of triplicate experiments. (D) Dimer formation of AR WT or AR P767A was determined using NanoBiT analysis. The arrow indicates the time‐point of addition of DHT or MeOH. The luminescence was monitored by Nivo (PerkinElmer), values of which are expressed as mean ± SEM of triplicate experiments. (E) Dimer formation of AR was determined using NanoBiT analysis. HEK293T shControl or shFKBP52 cells constitutively expressing FKBP52 WT and PPIase mutant (F67Y), which may also lack proper protein–protein interactions, were assayed as in Fig. 4A. Immunoblots of cells subjected to NanoBiT analysis are shown on the right. Cells were cultured in the presence of Dox for 2 days.

FKBPs possess PPIase domains that catalyze interconversion between the prolyl cis/trans conformations, thereby altering the conformation of their target proteins and acting as molecular switches. We next investigated whether multiple inhibitors of FKBPs affect AR dimerization in 293T cells. AR dimerization was found to be abrogated in the presence of the above‐mentioned FK506, FKBP51 PPIase inhibitor or SAFit2 (Fig. 6A,B). Furthermore, treatment with MJC13, which impairs FKBP52‐mediated AR function [29], also suppressed AR dimerization (Fig. 6C). These results are consistent with previous studies, which have shown that FK506 and MJC13 inhibit prostate cancer cell proliferation and AR activity [29, 30].

A reduction in AR dimerization in cells treated with the FKBP51 PPIase inhibitor suggests that FKBP51 may isomerize proline in AR. It was reported that AR Pro^767^ mutated to Ala remains a monomer, even in the presence of DHT [21]. We confirmed that the AR P767A mutant was defective in dimer formation compared to wild‐type AR (Fig. 6D). We next examined the effect of the PPIase activity of FKBP52 on AR dimerization. Overexpression of FKBP52 wild‐type (WT) substantially promoted AR dimerization upon DHT treatment (Fig. 6E), consistent with previous studies, which have shown that overexpression of FKBP52 increases AR transcriptional activity in yeast reporter assay [5]. Of note, the WT as well as the FKBP52 PPIase‐deficient mutant, F67Y, which may also lack proper protein–protein interactions, suppressed the defect in FKBP52 depletion. These results suggest that the PPIase activity of FKBP51, but not of FKBP52, plays a key role in AR dimerization upon ligand binding.

**Fig. 7:**
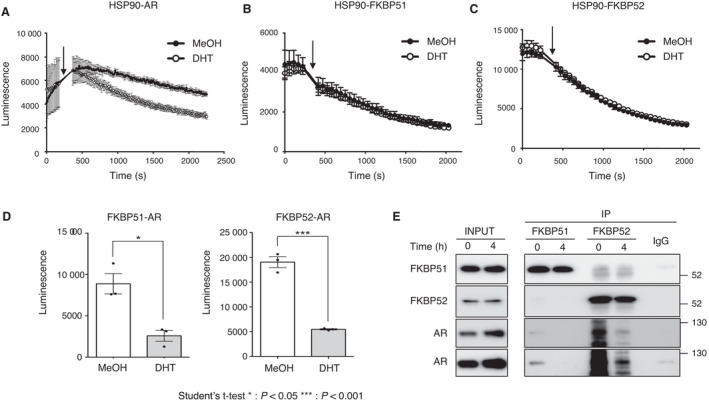
DHT induces dissociation of the AR‐FKBPs complex. (A–C) Proteination of the AR‐FKBPs between HSP90 and AR, FKBP51, or FKBP52 was determined using NanoBiT analysis in HEK293T cells. LgBiT‐HSP90 and SmBiT‐FKBP52, AR‐LgBiT, or LgBiT‐FKBP51 and SmBiT‐HSP90 expression vectors were cotransfected into HEK293T cells. The luminescence was monitored by GloMax^®^ Navigator (Promega) (A) or ARVO X4 (PerkinElmer) (B, C), values for which have been expressed as mean ± SEM of triplicate experiments. (D) Protein–interactions between AR and FKBP51 or FKBP52 were determined using NanoBiT analysis in HEK293T cells. AR‐LgBiT and FKBP‐SmBiT expression vectors were cotransfected into HEK293T cells. Cells were treated with methanol or DHT for 4 h. The bar graphs show the level of luminescence 15 min after addition of the Nano‐Glo^®^ Live Cell Reagent. The luminescence was monitored by ARVO X4 (PerkinElmer), values for which have been expressed as mean ± SEM of triplicate experiments. Statistical significance was determined using Student's *t*‐test. *P < 0.05 and ***P < 0.001. (E) Immunoprecipitation was carried out to detect the interaction of FKBP51 or FKBP52 with HSP90 and AR, with and without DHT, in 22Rv1 cells (*n* = 3).

For proper AR signaling, assembly and disassembly of protein–protein complexes need to be dynamically controlled. In particular, dissociation of a ligand‐bound active form of AR from the Hsp90‐containing complex is required for its transactivation [31], although the exact molecular events that govern this effect remain unclear. To examine the association of AR‐Hsp90‐FKBP complexes upon ligand binding, we investigated the dynamics of these interactions using NanoBiT assay. The interaction between Hsp90 and AR was attenuated as shown previously (Fig. 7A) [32], whereas the interactions between Hsp90 and FKBPs remained unchanged upon DHT treatment (Fig. 7B,C). Consistent with these results, upon DHT addition, there was a decrease in the interaction of FKBPs and AR as well (Fig. 7D). We confirmed the dissociation of FKBP‐AR upon DHT treatment using immunoprecipitation (Fig. 7E). These results suggest that promoting the dissociation of the steroid receptor from both chaperones and cochaperones triggers events such as nuclear translocation, dimer formation, or recognition of the DNA‐binding domain for the promoter sequences.

### 3.5 High expression of FKBP52 is related to prostate‐specific antigen recurrence rate in prostate cancer patients

**Fig. 8:**
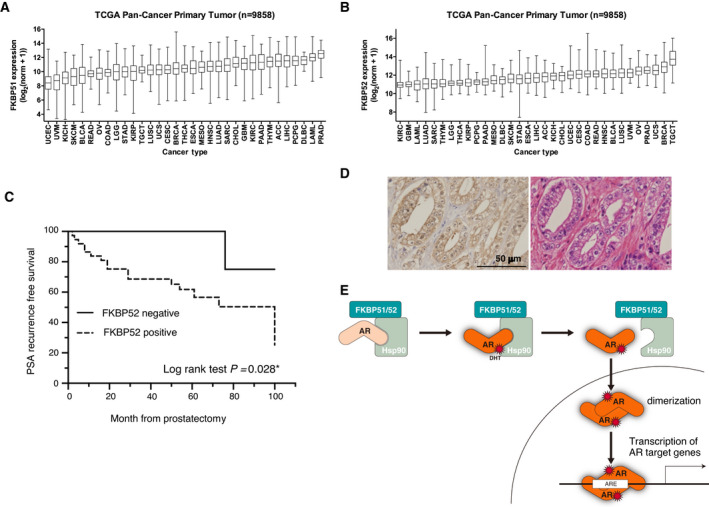
The PSA recurrence rate of FKBP52‐positive cases was significantly higher than that of negative cases. (A, B) mRNA expression levels of FKBP51 (A) and FKBP52 (B) in primary tumors of each cancer type in the TCGA Pan‐Cancer dataset. In the box plot, the central line reflects the median, while the borders of the boxes represent the interquartile range of the plotted data. The whiskers extend from minimum to maximum. (C) Kaplan–Meier curves for PSA recurrence‐free survival. The patients were divided into two groups according to the percentage of FKBP52 staining of the removed prostate tissue. *n* = 50 (37 FKBP52‐positive patients and 13 negative patients) log‐rank (Mantel–Cox) test: *P = *0.0228. (D) Representative case of positive expression of FKBP52 protein using immunohistochemistry (left) and H&E staining (right) in ×400 magnification. (E) Hypothesis model of FKBP51‐ and FKBP52‐mediated regulation of AR signaling. FKBP51 and FKBP52 interact with AR and facilitate AR dimer formation and prostate cancer progression.

We next examined the expression of FKBP51 and FKBP52 using The Cancer Genome Atlas (TCGA) Pan‐Cancer dataset (9858 samples) and found that the expression of FKBP51 was highest in prostate cancer, among various cancers (Fig. 8A). Similarly, the expression of FKBP52 was the 4th highest in prostate cancer, after tenosynovial giant cell tumor, breast carcinoma, and uterine carcinosarcomas (Fig. 8B).

Specimens obtained from prostate cancer patients were subjected to immunohistochemical (IHC) analysis with antibodies against FKBP52, following which the patients were classified into two categories based on FKBP52 expression. These groups of patients were tested for the blood level of prostate‐specific antigen (PSA), elevation of which is indicative of metastasis or recurrence after castration. PSA recurrence occurred in 75% of patients in the FKBP52‐positive group within 100 months, a significantly shorter time to recurrence than in the negative group (Fig. 8C). We performed a similar analysis for FKBP51 and found that expression of FKBP51 was not significantly correlated with the recurrence of prostate cancer after total resection. IHC analysis revealed that the expression of FKBP52 in the prostate cancer specimens was high in both the nucleus and the cytoplasm (Fig. 8D). These results indicated that FKBP51 and FKBP52 are highly expressed in prostate cancer and that high expression of FKBP52 is associated with a poor time until PSA recurrence in prostate cancer.

## 4 Discussion

In this study, we showed that the two immunophilins, FKBP51 and FKBP52, are known to be regulators of AR and are required for prostate cancer proliferation. The growth of prostate cancer is largely dependent on activation of AR. AR activity is regulated in a highly coordinated dynamic manner at multiple levels such as expression, androgen binding, nuclear translocation, homodimer formation, and DNA binding of AR. In the nucleus, an AR dimer binds to androgen‐responsive elements in the regulatory regions of its target genes [4]. Multiple reports have shown that FKBP52 promotes the activities of GR, MR, and AR, while FKBP51 attenuates GR and MR activity [33]. Indeed, FKBP51 overexpression was shown to increase AR transcriptional activity by promoting both the ligand binding to AR and AR assembly with Hsp90‐p23 complexes [34]. Previous studies have proposed that FKBP52 facilitates efficient transport of steroid receptors from the cytoplasm to the nucleus as a result of interaction with dynein [33]. However, our results showed that the translocation of AR to the nucleus was not affected by the depletion of either or both FKBP51 and FKBP52, suggesting that neither FKBP contributes to the translocation of AR.

Of note, the depletion of FKBP51 or FKBP52 reduced dimer formation, chromatin binding, phosphorylation, and transcriptional activity of AR, suggestive of defective AR signaling. Furthermore, the FKBP51 PPIase inhibitor and SAFit1/2 suppressed cell proliferation and colony‐forming ability, and inhibited ligand‐induced AR dimer formation. Treatment of prostate cancer cells with FK506, which binds to the FK1 domain of FKBPs, or with MJC13, an inhibitor of FKBP52‐AR signaling, also inhibited AR dimer formation.

FKBP51 was previously shown to attenuate the activity of Cdk4 by inhibiting the phosphorylation of Thr172 through the isomerization of Cdk4‐Pro173 in myocytes [35]. Based on this study, our results suggest the following regulatory model: In the absence of DHT, AR, HSP90, and FKBP51/52 form a complex in the cytoplasm. DHT may induce FKBP51 to isomerize proline of AR, a conformational change that leads to AR dimerization. Indeed, AR contains a proline‐rich region in the transcriptional activation domain. Furthermore, several mutants of AR that are causative of androgen insensitivity syndrome, including Pro^767^ mutated to Ala, remain as monomers even in the presence of DHT [21]. Consistent with the previous study, we found that the dimerization of AR‐P767A mutant was substantially attenuated compared with that of wild‐type AR. Thus, it is likely that FKBP51 may control AR conformation through isomerization at Pro^767^, which may trigger dimer formation. The enzymatic activity of FKBP52 is not involved in AR dimerization, suggesting that effects other than isomerization are important for AR dimerization. Dissociation of AR from the HSP90‐FKBP51/52 complex leads to nuclear translocation of AR, subsequent dimerization, and transcriptional activation of target genes (Fig. 8E).

FKBP51 and FKBP52 might be linked to the etiology of prostate cancer, given that the levels of FKBP51 and FKBP52 have been found to be elevated in human prostate cancer, compared to the noncancerous part of the prostate gland [36, 37, 38, 39]. Furthermore, expression of FKBP51 has been shown to be correlated with aggressiveness of cancers, such as glioma [40] and melanoma [41, 42]. In addition, elevated expression of FKBP52 correlates with tumor progression and predicts poor prognosis in individuals with breast cancer [43]. In the present study, we also demonstrated a correlation between FKBP52 expression and prognosis in prostate cancer patients. To our knowledge, this is the first study to show that FKBP52 expression is associated with the PSA recurrence of prostate cancer after total resection.

Collectively, our results suggest that inhibition of FKBP51 and FKBP52 activity might have a therapeutic effect on prostate cancer by abrogating AR dimer formation. In addition, dysregulation of FKBP51 or FKBP52 has been implicated in a variety of diseases, including stress‐related diseases and neurodegenerative disorders [28]. A better understanding of the molecular function of FKBP may, therefore, help in the treatment of these diseases.

## 5 Conclusion

Two immunophilins, FKBP51 and FKBP52, are known to be positive regulators of AR; however, the precise mechanism by which they control AR activity remains unclear. We found that both FKBPs were important for dimer formation and chromatin binding of AR. Furthermore, the PPIase activity of FKBP51 was found to be required for AR dimer formation and cancer cell growth. Of note, elevated expression of FKBP52 was associated with the PSA recurrence rate of prostate cancer. Collectively, these results suggest that FKBP51 and FKBP52 might be promising targets for prostate cancer treatment through the inhibition of AR dimer formation.

## Conflict of interest

The authors declare no conflict of interest.

## Author contributions

MS designed and coordinated this research. MS drafted the manuscript. KM, MH, MK, HM, SH, TM, YS, HM, KK, and HN carried out the experiments and data analysis. The authors have read and approved the final manuscript.

## Ethics statement

The studies involving human participants were reviewed and approved by Institute Research Ethics Committee of Yamaguchi University. Written informed consent was obtained from all the patients. The IRB approval number is H29‐132.

### Peer Review

The peer review history for this article is available at https://publons.com/publon/10.1002/1878‐0261.13030.

## Supporting information


**Fig. S1.** Synthesis of compound 6.
**Fig. S2.** Synthesis of FKBP inhibitor.
**Fig. S3.** FKBP51 and FKBP52 are essential for AR dimer formation.

## Data Availability

The data that support the findings of this study are available from the corresponding author upon reasonable request.
