# Supplementary material for: FKBP51 and FKBP52 regulate androgen receptor dimerization and proliferation in prostate cancer cells
Source: Mol Oncol. 2021 Jun 19;16(4):940–56. doi: 10.1002/1878-0261.13030 (PMC8847985; doi:10.1002/1878-0261.13030)
Supplement: Supplementary file 1 — Fig. S1. Synthesis of compound 6. Fig. S2. Synthesis of FKBP inhibitor. Fig. S3. FKBP51 and FKBP52 are essential for AR dimer formation. [file MOL2-16-940-s001.pdf]

Supplementary Fig S1

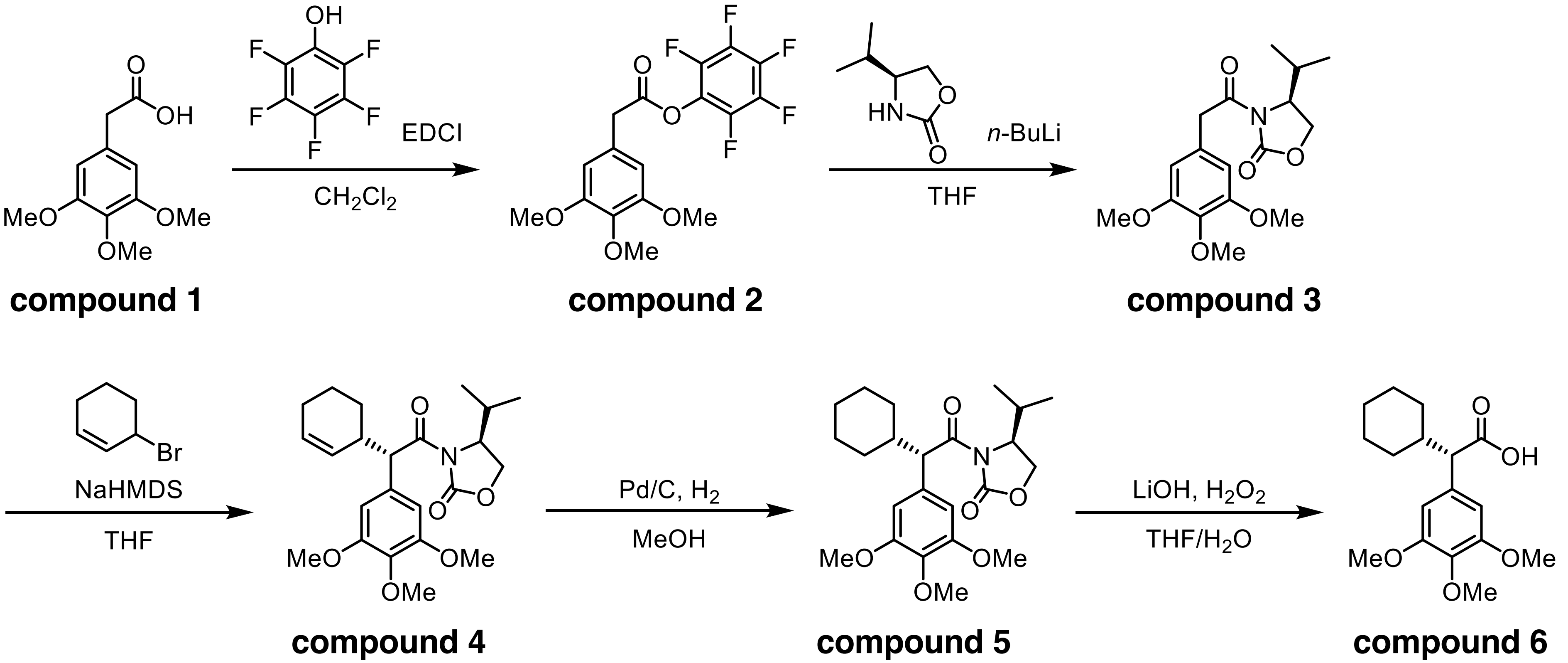

Supplementary Fig S1. Synthesis of compound 6

The FKBP51 PPIase inhibitor was synthesized in accordance with a previous report (Gaali et al., 2016), with slight modifications. Detailed procedures and spectral data are shown below.

Synthesis of 2,3,4,5,6-pentafluorophenyl 2-(3,4,5-trimethoxyphenyl)acetate (**compound 2**).

To a solution of 2-(3,4,5-trimethoxyphenyl)acetic acid (**compound 1**; 0.91 g, 4.0 mmol, 1.0 eq.) and EDCI (1.2 g, 6.0 mmol, 1.5 eq.) in CH_2Cl_2 (15 mL) was added 2,3,4,5,6-pentafluorophenol (1.1 g, 6.0 mmol, 1.5 eq.) in CH_2Cl_2 (7 mL). The reaction mixture was stirred at R.T. for 15 hr, then evaporated. The residue was purified by column chromatography on silica gel (eluent: *n*-hexane/AcOEt = 6/1 → 4/1) to obtain **compound 2** (1.4 g, 3.6 mmol), yield 89% as a white solid. ^{1}H NMR (500 MHz, $CDCl_3$): d 3.85 (s, 3H), 3.88 (s, 6H), 3.91 (s, 2H), 6.56 (s, 2H).

Synthesis of (*S*)-4-isopropyl-3-[2-(3,4,5-trimethoxyphenyl)acetyl]oxazolidin-2-one (**compound 3**).
To a solution of (*S*)-4-isopropyloxazolidin-2-one (0.26 g, 2.0 mmol, 1.0 eq.) in dry THF (10 mL) was added 1.6 M *n*-BuLi (1.3 mL, 2.0 mmol, 1.0 eq.) at – 78 ° C under Ar. The reaction mixture was stirred at – 78 ° C for 30 min, then added **compound 2** (0.79 g, 2.0 mmol, 1.0 eq.) in dry THF (4 mL). The reaction mixture was stirred for further 1 hr at – 78 ° C, then warmed up to R.T. and stirred for 20 hr. The reaction was quenched by adding sat. NH_4Cl aq. and extracted with CH_2Cl_2. The combined organic phase was washed with brine and dried over Na_2SO_4, then evaporated. The residue was purified by column chromatography on silica gel (eluent: *n*-hexane/AcOEt = 2/1 → 1/1) to obtain **compound 3** (0.30 g, 0.88 mmol), yield 44% as a light-yellow oil. ^{1}H NMR (500 MHz, $CDCl_3$): d 0.81 (d, 3H, *J* = 7.0 Hz), 0.89 (d, 3H, *J* = 7.0 Hz), 2.31–2.40 (m, 1H), 3.83 (s, 3H), 3.85 (s, 6H), 4.10–4.23 (m, 2H), 4.26–4.33 (m, 2H), 4.43–4.47 (m, 1H), 6.57 (s, 2H).

Synthesis of [(*S*)-2-(cyclohex-2-en-1-yl)-2-(3,4,5-trimethoxyphenyl)acetyl]-(*S*)-4-isopropyl-oxazolidin-2-one (**compound 4**)
To a solution of **compound 3** (0.30 g, 0.88 mmol, 1.0 eq.) in dry THF (8 mL) was added 1.0 M NaHMDS (0.88 mL, 0.88 mmol, 1.0 eq.) at – 78 ° C under Ar. The reaction mixture was stirred at – 78 ° C for 1 hr, then added 3-bromocyclohexene (0.10 mL, 0.88 mmol, 1.0 eq.) in dry THF (2 mL). The reaction mixture was stirred for further 1 hr at – 78 ° C, then warmed up to R.T. and stirred for 19 hr. The reaction was quenched by adding sat. NH_4Cl aq. and extracted with CH_2Cl_2. The organic phase was washed with brine and dried over Na_2SO_4, then evaporated. The residue was purified by column chromatography on silica gel (eluent: *n*-hexane/AcOEt = 3/1 → 2/1) to obtain **compound 4** (0.19 g, crude) as a light-yellow oil. ^{1}H NMR (500 MHz, $CDCl_3$): d 0.92–0.96 (m, 6H), 1.12–1.92 (m, 4H), 1.97–2.04 (m, 2H), 2.42–2.52 (m, 1H), 2.90–3.02 (m, 1H), 3.83 (s, 3H), 3.85 (s, 6H), 4.14–4.18 (m, 2H), 4.40–4.44 (m, 1H), 4.88–4.96 (m, 1H), 5.52–5.80 (m, 2H), 6.66–6.69 (m, 2H); MS (ESI$^+$): 440 $[M+Na]^+$.

Synthesis of [(*S*)-2-cyclohexyl-2-(3,4,5-trimethoxyphenyl)acetyl]-(*S*)-4-isopropyl-oxazolidin-2-one (**compound 5**)
To a solution of **compound 4** (0.19 g, crude) in MeOH (15 mL) was added 10% Pd/C (10 mg). The reaction mixture was stirred at R.T. for 18 hr under H_2, then filtered through Celite and evaporated to obtain crude product (0.19 g) as a pale yellow solid. The residue was purified by reversed-phase preparative HPLC (A:B = 40:60 to 0:100 (20 min), A: 0.1% formic acid MilliQ, B: 0.1% formic acid CH_3CN) to obtain **compound 5** (0.17 g, 0.40 mmol), yield 45% (in two steps) as a white solid. ^{1}H NMR (500 MHz, $CDCl_3$): d 0.78–0.88 (m, 1H), 0.91 (t, 6H, *J* = 7.2 Hz), 1.04–1.34 (m, 5H), 1.60–1.65 (m, 2H), 1.68–1.77 (m, 2H), 2.06–2.15 (m, 1H), 2.39–2.46 (m, 1H), 3.81 (s, 3H), 3.84 (s, 6H), 4.14 (d, 2H, *J* = 5.7 Hz), 4.37–4.42 (m, 1H), 4.82 (m, 1H, *J* = 10.6 Hz), 6.63 (s, 2H); MS (ESI$^+$): 442 $[M+Na]^+$.

Synthesis of (*S*)-2-cyclohexyl-2-(3,4,5-trimethoxyphenyl)acetic acid (**compound 6**)
To a solution of **compound 5** (0.17 g, 0.40 mmol, 1.0 eq.) in THF/H_2O (5 mL/3 mL) was added LiOH•H_2O (0.034 g, 0.80 mmol, 2.0 eq.) and 8.8 M H_2O_2 (0.91 mL, 8.0 mmol, 20 eq.). The reaction mixture was stirred at R.T. for 24 hr, then quenched by adding 1.5 M Na_2SO_3 aq. The solution was diluted with brine and washed with CH_2Cl_2, then acidified with 2 N HCl aq. The solution was extracted with CH_2Cl_2, and combined organic phase was washed with brine and dried over Na_2SO_4, then evaporated to obtain **compound 6** (0.12 g, 0.39 mmol), yield 98% as a colorless oil. ^{1}H NMR (500 MHz, $CDCl_3$): d 0.70–0.80 (m, 1H), 1.02–1.22 (m, 3H), 1.24–1.42 (m, 2H), 1.60–1.80 (m, 3H), 1.84–2.00 (m, 2H), 3.12 (d, 1H, *J* = 10.7 Hz), 3.82 (s, 3H), 3.85 (s, 6H), 6.54 (s, 2H); ^{13}C NMR (125 MHz, $CDCl_3$): d 25.9, 26.2, 30.3, 31.9, 56.1, 58.9, 60.8, 105.5, 132.8, 137.2, 153.1, 179.2; MS (ESI$^+$): 331 $[M+Na]^+$.

Supplementary Fig S2

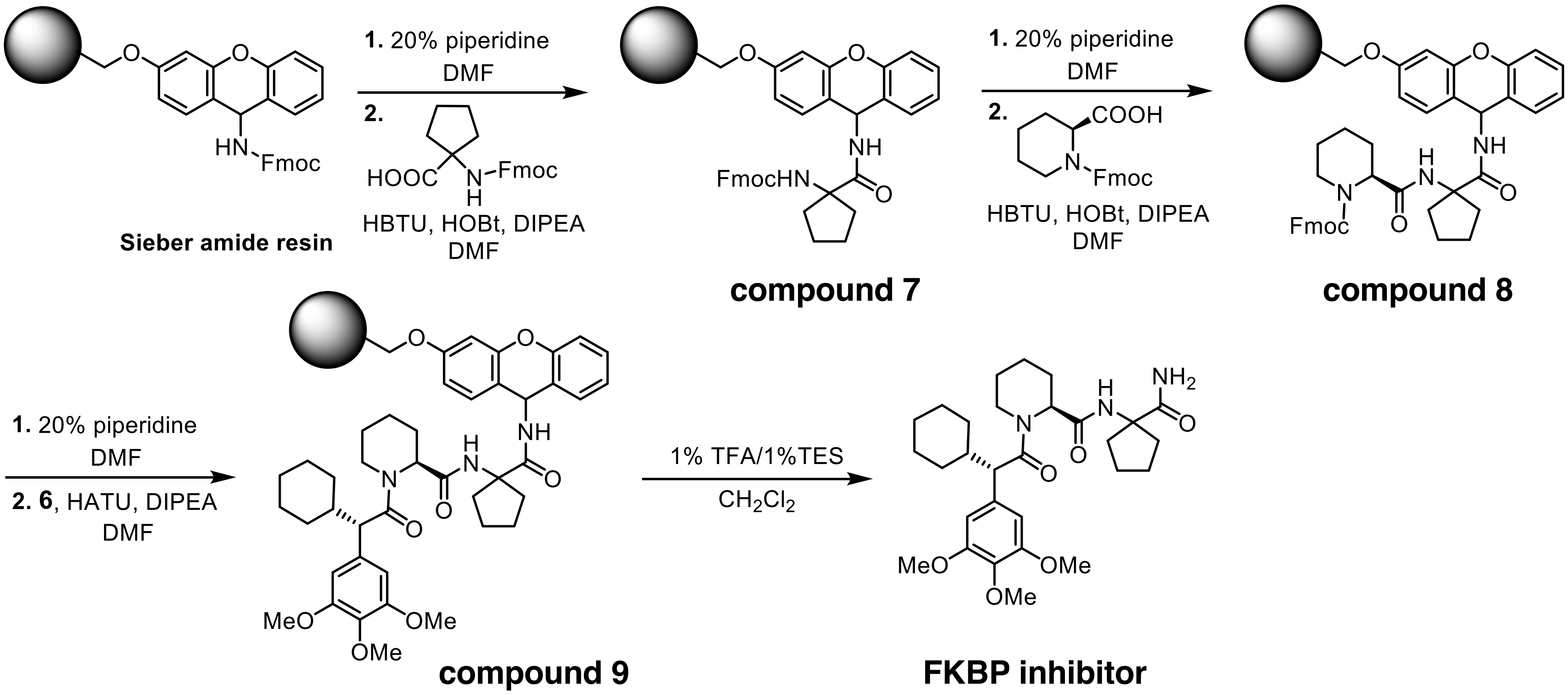

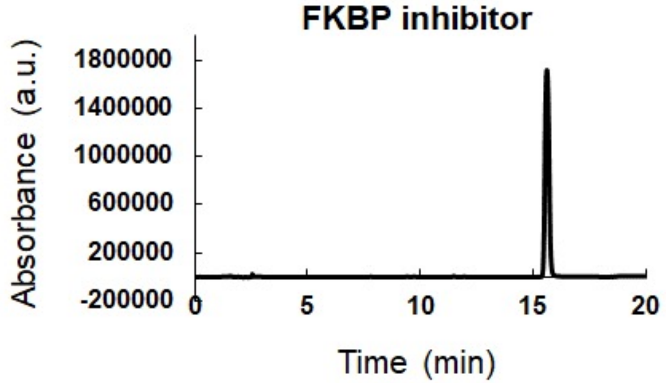

Supplementary Fig S2. Synthesis of FKBP inhibitor

Sieber amide resin (0.074 g, 0.040 mmol) was treated with 20% piperidine in DMF (2 mL) twice for 2 min and 8 min, then washed with DMF. To the resin was added Fmoc-cycloleucine-OH (0.070 g, 0.20 mmol, 5.0 eq.), HBTU (0.076 g, 0.20 mmol, 5.0 eq.), HOBt (0.031 g, 0.20 mmol, 5.0 eq.) and DIPEA (0.070 mL, 0.40 mmol, 10 eq.) in DMF (2 mL). The mixture was stirred at R.T. for 11 hr, then washed with DMF. Fmoc-deprotection and washing was repeated as described above. To the resin was added Fmoc-Pip-OH (0.070 g, 0.20 mmol, 5.0 eq.), HBTU (0.076 g, 0.20 mmol, 5.0 eq.), HOBt (0.031 g, 0.20 mmol, 5.0 eq.) and DIPEA (0.070 mL, 0.40 mmol, 10 eq.) in DMF (2 mL). The mixture was stirred at R.T. for 2 hr, then washed with DMF. Deprotection and washing was performed as above, followed by the addition of **compound 6** (0.026 g, 0.085 mmol, 2.0 eq.), HATU (0.032 g, 0.085 mmol, 2.0 eq.) and DIPEA (0.030 mL, 0.17 mmol, 4.0 eq.) in DMF (2 mL). The mixture was stirred at R.T. for 18 hr, then washed with DMF, MeOH, CH_2Cl_2 and Et_2O and dried *in vacuo*. Crude **FKBP inhibitor** was cleaved from the resin by using 1% TFA/1% triethylsilane (TES)/CH_2Cl_2 (2 mL $\times$ 5). The cleaving solution was neutralized with sat. $NaHCO_3$ aq. and the product was extracted with CH_2Cl_2 and washed with brine and dried over Na_2SO_4, then evaporated to obtain crude **FKBP inhibitor** (0.023 g). The residue was purified by reversed-phase preparative HPLC (A:B = 50:50 to 0:100 (20 min), A: 0.1% formic acid MilliQ, B: 0.1% formic acid CH_3CN) to obtain pure **FKBP inhibitor** (0.020 g, 0.38 mmol), yield 96% as a colorless amorphous. ^{1}H NMR (500 MHz, $CDCl_3$): d 0.69–0.80 (m, 1H), 0.84–0.96 (m, 1H), 1.09–1.41 (m, 7H), 1.48–1.78 (m, 11H), 1.80–1.88 (m, 1H), 2.03–2.21 (m, 3H), 2.24–2.32 (m, 1H), 2.85–2.94 (m, 1H), 3.40 (d, 1H, J = 10.3 Hz), 3.80 (s, 3H), 3.85 (s, 6H), 4.04–4.12 (m, 1H), 5.06–5.12 (m, 1H), 5.48 (brs, 1H), 6.54 (s, 2H), 6.59 (brs, 1H), 8.01 (brs, 1H); ^{13}C NMR (125 MHz, $CDCl_3$): d 20.3, 23.7, 23.9, 24.9, 25.5, 26.0, 26.0, 26.4, 30.4, 32.6, 36.3, 37.3, 41.3, 43.9, 53.0, 55.1, 56.3, 60.8, 67.0, 105.2, 133.4, 137.3, 153.5, 171.3, 174.0, 176.0; HRMS (ESI$^+$): calcd: 552.3050; found: 552.3049 $[M+Na]^+$ (– 0.14 ppm). HPLC purity (at 254 nm): 97.1%, t_R = 15.6 min (HPLC condition: A:B = 80:20 to 0:100 (20 min), A: 0.1% formic acid MilliQ, B: 0.1% formic acid CH_3CN.).

1H- and 13C-NMR spectra of the final product completely matched the report.

Supplementary Fig S3

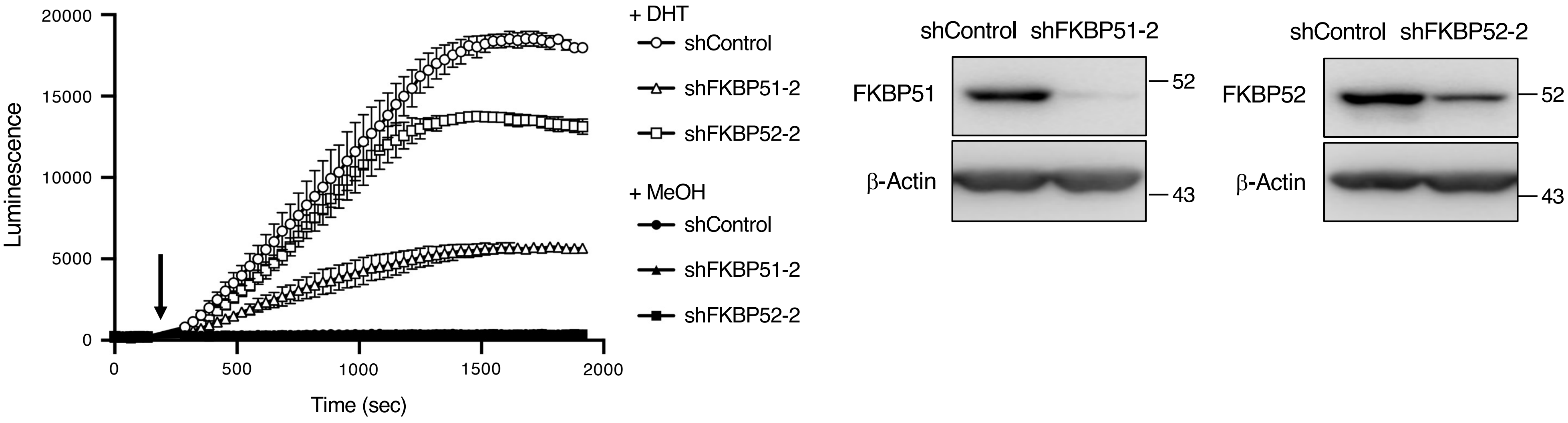

Supplementary Fig S3. FKBP51 and FKBP52 are essential for AR dimer formation

Dimer formation of AR was determined using NanoBiT analysis. HEK293T shControl, shFKBP51-2, or shFKBP52-2 cells were assayed as Figure 3A (left). Knockdown efficiency was confirmed by immunoblotting (right).
